# Male and Female Cat Fleas (*Ctenocephalides felis*) Differ in Bacterial Control, Survival, and Circulating Hemocyte Responses Following Systemic Challenge

**DOI:** 10.3390/insects17090952

**Published:** 2026-09-12

**Authors:** Ashley Lynch, Margalit Mauricio, Yuriani Palomino, Lisa D. Brown

**Affiliations:** Department of Biology, Georgia Southern University, 4324 Old Register Rd., Statesboro, GA 30460, USA

**Keywords:** fleas, hemocytes, phagocytosis, sex differences, systemic infection, bacterial infection

## Abstract

Although both male and female cat fleas (*Ctenocephalides felis*) consume vertebrate blood throughout adulthood, previous studies of flea immunity have focused primarily on females, leaving whether systemic immune responses differ between the sexes largely unknown. To address this knowledge gap, we compared bacterial persistence, survival, circulating hemocyte abundance, and phagocytic activity in male and female *C. felis* following microinjection of *Escherichia coli* into the hemocoel. Females exhibited significantly lower infection prevalence and bacterial burdens than males 24 h post-challenge. Consistent with these differences, bacterial infection reduced survival in both sexes but had a substantially greater effect on males, while sterile injury alone significantly reduced male survival but had little effect on females. Across all treatment groups, females possessed significantly higher circulating hemocyte abundance than males and were the only sex to exhibit an increase in circulating hemocyte numbers following injury or bacterial challenge. Additionally, a significantly greater proportion of female hemocytes participated in phagocytosis, although the number of bacteria internalized per hemocyte did not differ between the sexes. Together, these findings demonstrate sex-specific differences in the systemic antibacterial responses of *C. felis*, with females exhibiting lower bacterial burdens, greater survival, higher circulating hemocyte abundance, and a greater proportion of phagocytic hemocytes than males. These results identify sex as a biological variable influencing systemic immune responses in cat fleas and provide a foundation for determining whether similar differences occur following natural routes of microbial exposure.

## Simple Summary

Fleas transmit several microorganisms that cause disease in humans and animals, yet relatively little is known about how their immune systems respond to infection. Previous studies of cat flea immunity have focused primarily on females, leaving unanswered the question of whether males and females respond similarly to invading microorganisms. In this study, we compared the responses of male and female cat fleas after bacteria were experimentally introduced directly into their body cavity. Female fleas maintained lower levels of bacterial infection and survived better than males. Females also had higher circulating immune cell counts and increased these counts following injury and bacterial challenge, whereas males did not. In addition, a greater proportion of female immune cells captured and consumed bacteria, although individual immune cells from males and females consumed similar numbers of bacteria. These findings demonstrate that male and female cat fleas differ in their responses to experimental bacterial infection. Understanding variation in immune function between the sexes provides new insight into flea biology and establishes a foundation for future studies examining whether similar differences occur when fleas encounter disease-causing microorganisms through natural routes of infection.

## 1. Introduction

Despite their importance as vectors of numerous zoonotic pathogens, the mechanisms by which fleas (Order Siphonaptera) respond to microbial infection remain poorly understood compared with those of other hematophagous arthropods [1,2]. Like all insects, fleas rely exclusively on innate immunity, which consists of coordinated cellular and humoral responses that detect and combat invading microorganisms [3,4,5]. Cellular immunity is mediated primarily by hemocytes, which eliminate pathogens through phagocytosis, encapsulation, and nodulation, while humoral defenses involve activation of conserved immune signaling pathways that stimulate the production of antimicrobial peptides, reactive oxygen species, and other soluble immune effectors. Much of the current understanding of these defenses comes from studies of *Drosophila melanogaster*, where genetic and molecular approaches have provided a framework for investigating innate immunity in less well-characterized insects [5]. Recent studies have begun to characterize flea immune defenses, including the involvement of conserved immune signaling pathways such as IMD in antibacterial responses [6,7,8,9,10,11,12,13,14,15], yet the biological factors that influence infection outcomes remain largely unexplored.

Sex-specific differences in immune function have been documented across a broad range of animal taxa and are thought to contribute to variation in susceptibility and resistance to infection [16]. Traditionally, females have been considered the more immunocompetent sex because selection favors prolonged survival and sustained reproductive output, whereas males are expected to allocate proportionally more resources toward reproductive traits than immune defense [17,18,19,20]. However, accumulating evidence indicates that immune sexual dimorphism is neither universal nor consistently female-biased. In insects, patterns of sex-biased immunity are often condition- and pathogen-dependent, and the mechanisms responsible remain poorly understood (extensively reviewed in [16,21]). One factor proposed to influence these differences is feeding ecology, as males and females of many insect species exploit different nutritional resources and consequently experience distinct microbial environments and immune challenges [22]. This hypothesis is particularly relevant in hematophagous insects, where sex-specific blood-feeding behavior varies considerably among taxa. For example, in mosquitoes (*Aedes aegypti* and *Anopheles aquasalis*) and sand flies (*Lutzomyia longipalpis*), where males feed exclusively on plant nectar and females feed on both plant nectar and vertebrate blood, males exhibit higher basal expression of several conserved immune genes involved in systemic humoral immunity [23]. Conversely, in the kissing bug *Rhodnius prolixus*, where both sexes are obligate blood feeders, females exhibit higher basal expression of these same immune genes than males [23]. These contrasting patterns suggest that differences in feeding ecology may contribute to the evolution of immune sexual dimorphism.

To date, systemic innate immune responses to bacterial infection have been investigated in only one flea species, the cat flea, *Ctenocephalides felis*. Using model Gram-positive and Gram-negative bacteria, Muñoz et al. [10] demonstrated that both cellular and humoral immune defenses contribute to antibacterial immunity, with microbial challenge eliciting increased circulating hemocyte abundance and robust phagocytic activity. Moreover, experimental impairment of phagocytosis led to significantly higher bacterial burdens, thereby establishing hemocyte-mediated phagocytosis as an important component of the systemic antibacterial response. However, this study was conducted exclusively on female fleas, leaving it unknown whether males and females differ in their systemic immune responses. Therefore, this study aimed to determine whether male and female *C. felis* differ in their responses to systemic bacterial challenge.

## 2. Materials and Methods

### 2.1. Flea Maintenance

Adult cat fleas (*C. felis*) were obtained from a laboratory colony maintained at Georgia Southern University or purchased from Ecto Services, Inc. (Henderson, NC, USA). Fleas were maintained on defibrinated bovine blood (HemoStat Laboratories, Dixon, CA, USA) using an artificial membrane feeding system as previously described [6]. All experiments were performed using blood-fed adult fleas between 2 and 3 days post-emergence. Male and female fleas were distinguished by external reproductive morphology under a dissecting microscope based on the terminal abdominal segments and genitalia [24]. Males and females were housed together and provided blood ad libitum, except when separated by sex for experimental manipulation and sample collection.

### 2.2. Bacterial Culture and Flea Infection

Ampicillin-resistant, green fluorescent protein (GFP)-expressing *Escherichia coli* (ATCC^®^ 25922GFP, Manassas, VA, USA; *E. coli*) was cultured and prepared for flea infection as previously described by Muñoz et al. [10]. Briefly, bacterial cultures were grown overnight in tryptic soy broth (TSB) supplemented with ampicillin (100 µg/mL) at 37 °C with shaking (350 rpm) and standardized to an optical density of OD_600_ = 5.0 using a BioPhotometer D30 (Eppendorf AG, Hamburg, Germany) prior to injection.

Fleas were cold-anesthetized and injected into the thorax using a Nanoject III Auto-Nanoliter Injector (Drummond Scientific Company, Broomall, PA, USA). Each flea received 69 nL of either *E. coli* suspended in TSB or sterile TSB alone as an injury control. Additional fleas were cold-anesthetized but not injected to serve as naïve controls. Fleas that died within 1 h of treatment were excluded from the analysis to minimize mortality associated with handling or injection. Sample sizes reported for each experiment represent the number of surviving individuals in the experimental cohort after the 1 h exclusion period. The number of individuals excluded during this initial period was not recorded. To verify the inoculation dose, a subset of fleas was homogenized immediately after injection, plated on tryptic soy agar (TSA) containing ampicillin, incubated overnight at 37 °C, and colony-forming units (CFUs) were enumerated. Each experimental endpoint was evaluated in separate experiments using independently prepared cohorts of fleas and bacterial cultures.

### 2.3. Bacterial Persistence

To quantify bacterial persistence, male and female fleas were injected with *E. coli* as described above. Twenty-four hours after infection, individual fleas were homogenized in 200 µL phosphate-buffered saline (PBS), serially diluted, and plated on TSA containing ampicillin. Following overnight incubation at 37 °C, CFUs were enumerated. Infection was quantified as (1) prevalence, defined as the proportion of fleas yielding detectable *E. coli*, and (2) intensity, defined as the number of CFUs recovered from infected individuals. Three independent biological replicates were performed, each consisting of 30 females and 30 males (*n* = 90 fleas per sex).

### 2.4. Survival Analysis

To determine whether bacterial infection differentially affected survival, male and female fleas were assigned to one of three treatment groups: naïve, TSB-injected (injury), or *E. coli*-injected. Following treatment, male and female fleas were maintained together in the same feeding chambers under standard colony conditions and provided defibrinated bovine blood ad libitum using the artificial membrane feeding system described above. Survival was monitored daily for 5 days by recording the number of live and dead fleas in each treatment group, and dead fleas were removed after each daily observation. Three independent biological replicates were conducted, each consisting of 30 females and 30 males per treatment (*n* = 90 per sex per treatment).

### 2.5. Circulating Hemocyte Counts

Male and female fleas were assigned to the naïve, TSB-injected, or *E. coli*-injected treatment groups. Twenty-four hours after treatment, circulating hemocytes were collected by perfusion as previously described by Muñoz et al. [10]. Briefly, an incision was made across the penultimate abdominal segment, and approximately 200 μL of PBS was injected into the thorax. Although the osmolarity of *C. felis* hemolymph has not been characterized and PBS was not independently validated for hemocyte viability in the present study, this method previously yielded morphologically intact flea hemocytes suitable for quantitative and functional analyses [10]. Hemolymph exiting through the abdominal incision was collected on glass slides, allowed to adhere for 20 min at room temperature, fixed, and stained with Hema 3 (Fisher Scientific, Pittsburgh, PA, USA). Total circulating hemocytes were counted under bright-field illumination at 400× magnification. Three independent biological replicates were performed, each consisting of 20 females and 20 males per treatment (*n* = 60 fleas per sex per treatment).

### 2.6. Phagocytosis Assay

To evaluate phagocytic activity, male and female fleas were injected with *E. coli* as described above. One hour after infection, circulating hemocytes were collected by perfusion and stained with Hema 3 [10]. For each flea, the first 100 hemocytes observed were examined microscopically, and the number of intracellular bacteria was recorded. These observations were used to calculate the phagocytic index (percentage of hemocytes containing bacteria) and phagocytic capacity (mean number of bacteria per hemocyte). Three independent biological replicates were performed, each consisting of 10 females and 10 males (*n* = 30 fleas per sex).

### 2.7. Statistical Analysis

Data from independent biological replicates were pooled for analysis in GraphPad Prism version 10 (GraphPad Software, San Diego, CA, USA). Differences in bacterial prevalence, infection intensity, and phagocytic capacity were analyzed using the Mann–Whitney test. The phagocytic index was compared using Welch’s *t*-test. Circulating hemocyte counts were analyzed by two-way ANOVA with treatment and sex as fixed factors, followed by Tukey’s multiple-comparison test. Survival curves were compared using the Logrank (Mantel–Cox) test. Statistical significance was defined as *p* ≤ 0.05. For survival analyses, Bonferroni correction was applied to account for multiple pairwise comparisons, resulting in an adjusted significance threshold of *p* < 0.0167.

## 3. Results

### 3.1. Females Exhibit Lower Bacterial Prevalence and Burden than Males Following Systemic Challenge

**Figure 1:**
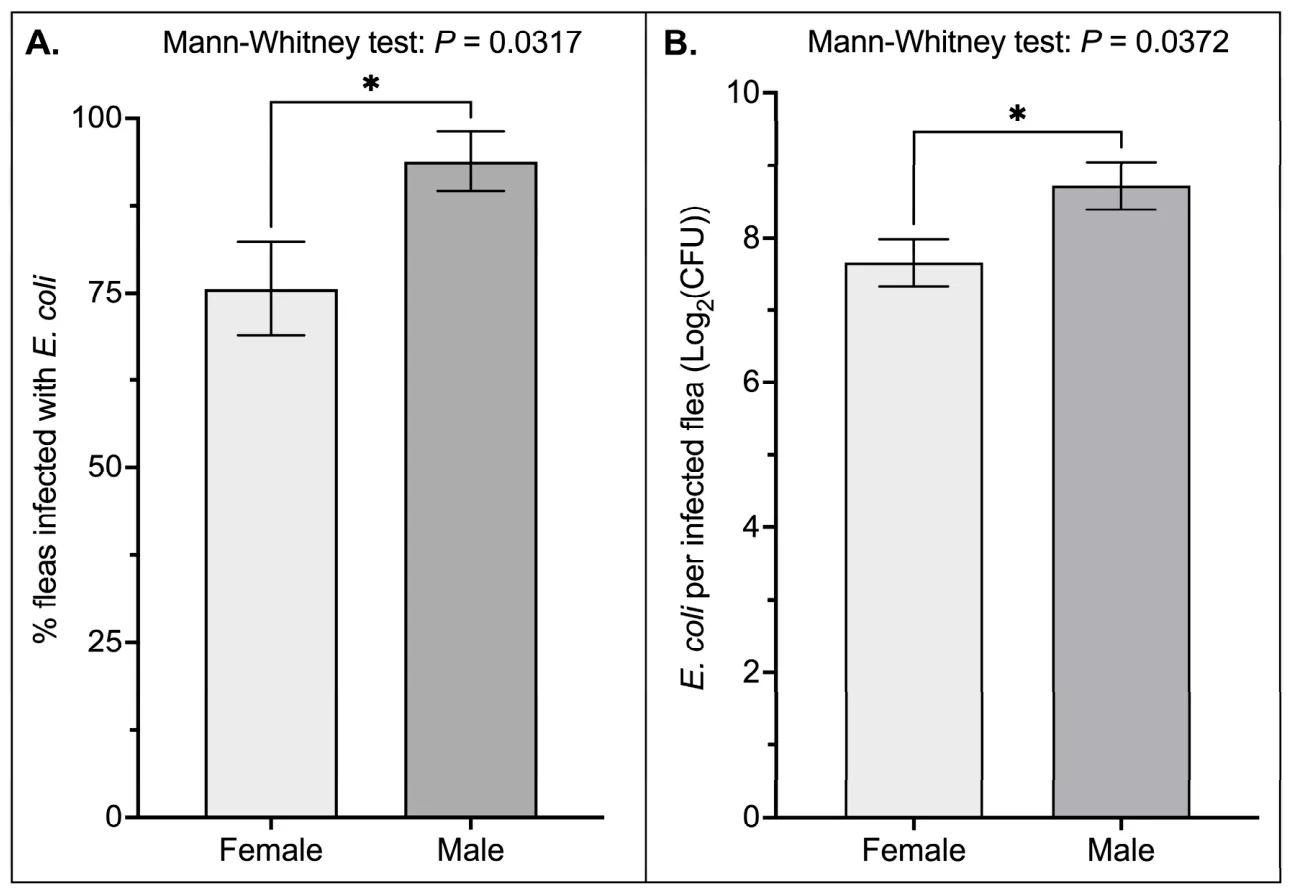
Measurement of bacterial infection prevalence and intensity in male and female *Ctenocephalides felis* following systemic *E. coli* infection at 24 h post-infection. (**A**) Mean percentage of fleas infected with *E. coli* (infection prevalence). (**B**) Mean number of *E. coli* per infected flea (infection intensity). Error bars represent the standard error of the mean (SEM). Three independent experiments were performed, each containing 30 females and 30 males (*n* = 90 fleas per sex). Statistical comparisons were performed using the Mann–Whitney test, with significance defined as *p* ≤ 0.05. Asterisks denote statistical significance: * *p* ≤ 0.05.

To determine whether bacterial persistence differed between the sexes, male and female fleas were injected with *E. coli* into the hemocoel, and bacterial infection was quantified 24 h post-injection by measuring infection prevalence (the proportion of infected fleas) and infection intensity (the mean number of bacteria per infected flea). Males exhibited significantly greater infection prevalence than females (Figure 1A; Mann–Whitney, *p* = 0.0317), with 24% more males remaining infected 24 h after challenge. Likewise, infection intensity was significantly greater in males than females (Figure 1B; Mann–Whitney, *p* = 0.0372), with infected males harboring 12% more bacteria than infected females. Collectively, these findings demonstrate that female fleas exhibit lower bacterial prevalence and bacterial burdens than males 24 h after systemic challenge.

### 3.2. Females Exhibit Greater Survival Following Systemic Bacterial Infection than Males

**Figure 2:**
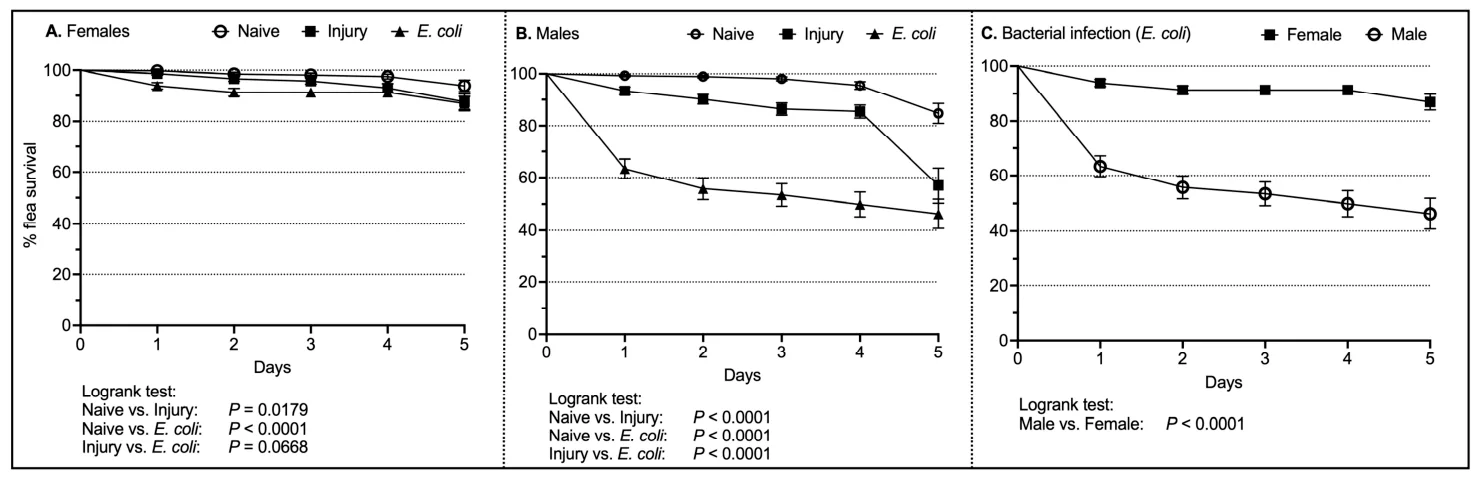
Survival of female and male *Ctenocephalides felis*. Fleas were left unmanipulated (naïve), injured, or infected with *Escherichia coli*, and survival was monitored daily for 5 days. (**A**) Survival of female fleas. (**B**) Survival of male fleas. (**C**) Direct comparison of *E. coli*-infected female and male fleas. Data from three independent experiments were combined, and error bars represent the standard error of the mean (SEM). Three independent biological replicates were conducted, each consisting of 30 females and 30 males per treatment (*n* = 90 per sex per treatment). Statistical comparisons were performed using the log-rank (Mantel–Cox) test. Because panels A and B each compared three treatment groups (naïve, injury, and *E. coli*), a Bonferroni correction for multiple comparisons was applied, with statistical significance defined as *p* ≤ 0.0167. Panel C compared only two groups (*E. coli*-infected females and males) and was considered significant at *p* ≤ 0.05.

To determine whether bacterial infection differentially affected survival, male and female fleas were assigned to one of three treatment groups (naïve, injury, or *E. coli*) and survival was monitored for 5 days. Relative to naïve controls, *E. coli* infection significantly reduced survival in both females (Figure 2A; Logrank test, *p* < 0.0001) and males (Figure 2B; Logrank test, *p* < 0.0001). In females, survival did not differ between the naïve and injury control groups (Figure 2A; Logrank test, *p* = 0.0179) or between the injury control and *E. coli*-infected groups (Figure 2A; Logrank test, *p* = 0.0668). In contrast, sterile injury alone significantly reduced survival in males compared with naïve controls (Figure 2B; Logrank test, *p* < 0.0001), although *E. coli* infection caused the greatest reduction in survival (Figure 2B; Logrank test, *p* < 0.0001).

Direct comparison of survival following *E. coli* infection revealed that females survived significantly longer than males (Figure 2C; Logrank test, *p* < 0.0001). By day 5 post-infection, 87% of *E. coli*-infected females remained alive compared with only 46% of infected males. In contrast, survival of naïve fleas was 94% in females and 85% in males, while sterile injury reduced male survival to 57% but had little effect on female survival (88%). Collectively, these findings demonstrate that systemic bacterial infection has a substantially greater impact on male survival than female survival.

### 3.3. Females Have Higher Circulating Hemocyte Counts than Males, and Only Females Increase Hemocyte Abundance Following Injury and Bacterial Challenge

**Figure 3:**
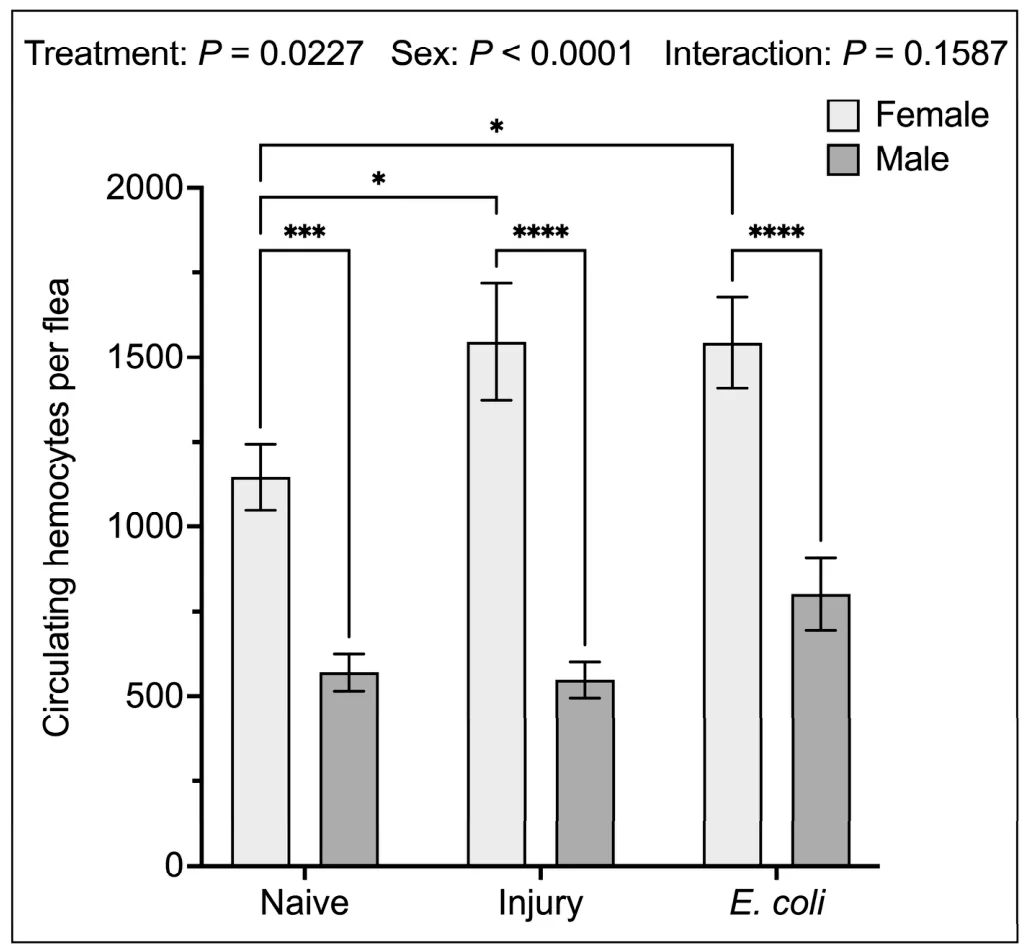
Total number of circulating hemocytes in female and male *Ctenocephalides felis*. Fleas were left unmanipulated (naïve), injured, or infected with *Escherichia coli*, and circulating hemocytes were counted 24 h later. Column heights represent the mean number of circulating hemocytes from three independent experiments, and error bars represent the standard error of the mean (SEM). Three independent experiments were performed, each containing 20 females and 20 males per treatment (*n* = 60 fleas per sex per treatment). Data were analyzed by two-way ANOVA followed by Tukey’s multiple comparisons test. Differences were considered significant at *p* ≤ 0.05. Asterisks denote statistical significance: * *p* ≤ 0.05, *** *p* ≤ 0.001, **** *p* ≤ 0.0001.

Circulating hemocytes are a major component of the insect cellular immune response and can influence the ability to eliminate invading microorganisms. To determine whether sex-specific differences in systemic antibacterial responses were associated with differences in circulating hemocyte abundance, male and female fleas were assigned to naïve, injury, or *E. coli*-infected treatment groups, and circulating hemocytes were quantified 24 h later. Across all treatment groups, females exhibited significantly higher circulating hemocyte counts than males (Figure 3; Naïve: Female vs. Male, Tukey’s, *p* = 0.0001; Injury: Female vs. Male, Tukey’s, *p* < 0.0001; *E. coli*: Female vs. Male, Tukey’s, *p* < 0.0001). In particular, naïve and *E. coli*-infected females possessed 101% and 93% more circulating hemocytes, respectively, than their male counterparts. Within females, both sterile injury and *E. coli* infection significantly increased circulating hemocyte counts relative to naïve controls (Figure 3; Naïve vs. Injury, Tukey’s, *p* = 0.0265; Naïve vs. *E. coli*, Tukey’s, *p* = 0.0343), corresponding to a 35% increase in circulating hemocyte abundance following either treatment. In contrast, circulating hemocyte counts did not differ among treatment groups in males. Collectively, these findings demonstrate that female fleas maintain a larger circulating hemocyte population than males and increase circulating hemocyte abundance following injury and bacterial challenge, whereas males exhibit no detectable change.

### 3.4. Females Exhibit a Higher Proportion of Phagocytic Hemocytes than Males, Whereas Bacterial Uptake per Hemocyte Is Comparable Between the Sexes

**Figure 4:**
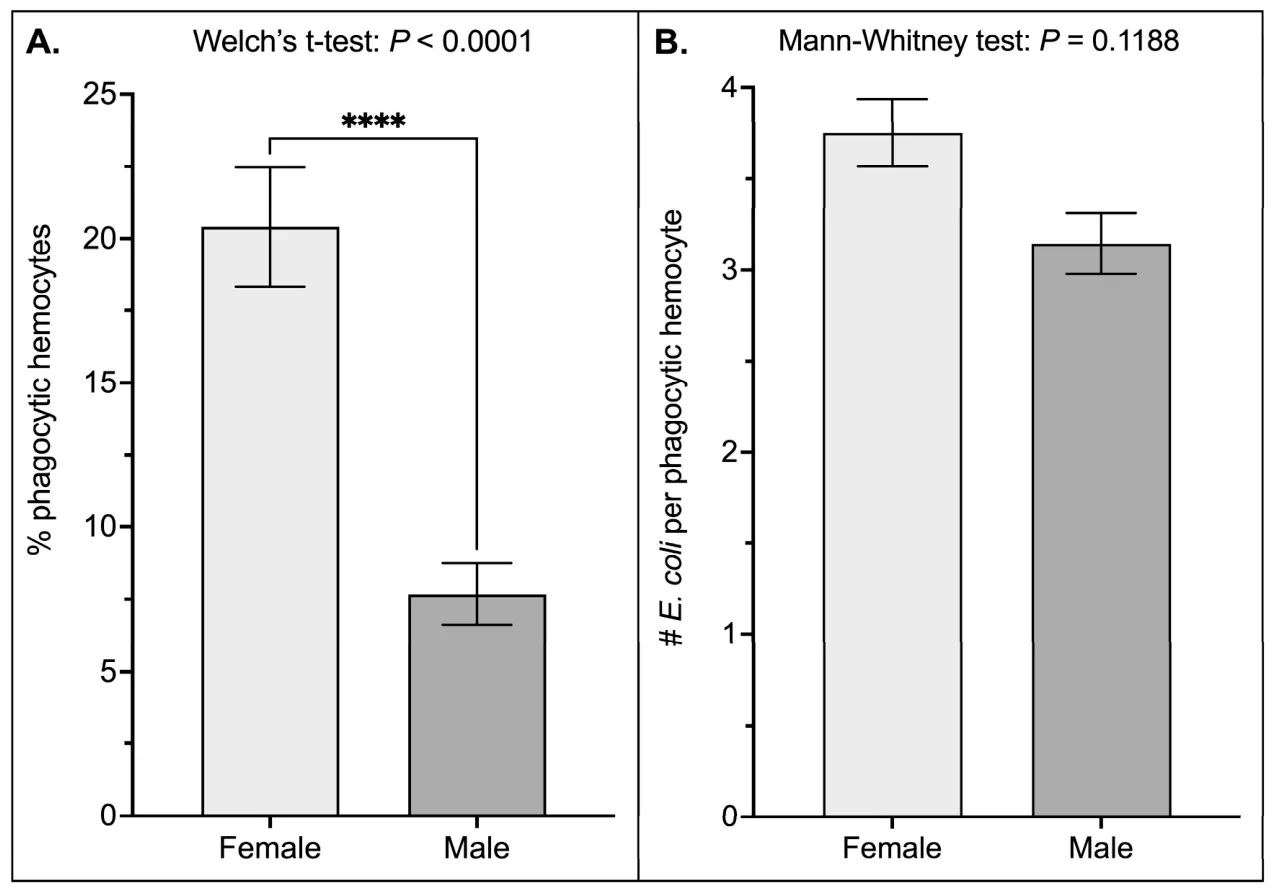
Phagocytic activity of circulating hemocytes in female and male *Ctenocephalides felis*. (**A**) Mean percentage of circulating hemocytes that engaged in phagocytosis. (**B**) Mean number of *Escherichia coli* per phagocytic hemocyte (phagocytic capacity). Error bars represent the standard error of the mean (SEM). Three independent experiments were performed, each containing 10 females and 10 males (*n* = 30 fleas per sex). Data were analyzed using Welch’s *t*-test (**A**) and the Mann–Whitney test (**B**). Differences were considered significant at *p* ≤ 0.05. Asterisks denote statistical significance: **** *p* ≤ 0.0001.

Because hemocytes mediate bacterial burden through phagocytosis, sex-specific differences in systemic antibacterial responses may result not only from variation in circulating hemocyte abundance but also from differences in the proportion or activity of hemocytes participating in phagocytosis. To determine whether phagocytic activity differs between male and female fleas, individuals of both sexes were injected with *E. coli* into the hemocoel, and phagocytosis was quantified 1 h later. The phagocytic index, defined as the percentage of hemocytes containing internalized bacteria, was significantly greater in females than in males (Figure 4A; Welch’s *t*-test, *p* < 0.0001). Specifically, females exhibited a 150% higher phagocytic index than males. In contrast, phagocytic capacity, defined as the number of bacteria internalized per hemocyte, did not differ between the sexes (Figure 4B; Mann–Whitney test, *p* = 0.1188). Individual hemocytes internalized between 1 and 18 *E. coli* cells in females and between 1 and 12 cells in males. Collectively, these findings indicate that enhanced bacterial uptake by female fleas is driven by a greater proportion of hemocytes participating in phagocytosis rather than by increased phagocytic capacity of individual hemocytes.

## 4. Discussion

The present study demonstrates that male and female *C. felis* differ in their responses to experimental systemic bacterial challenge. Following introduction of an equivalent *E. coli* inoculum directly into the hemocoel, females exhibited lower bacterial prevalence and burdens, greater survival, higher circulating hemocyte abundance, and a greater proportion of phagocytic hemocytes than males. These findings provide evidence of sex-specific variation in systemic antibacterial responses in *C. felis* and lay the groundwork for future studies examining whether comparable differences occur when flea-borne pathogens are acquired via an infectious blood meal.

One of the clearest differences between male and female *C. felis* was the lower prevalence and intensity of bacterial infection observed in females 24 h after systemic challenge. Because males and females received identical doses of *E. coli*, these differences reflect greater control of the experimentally introduced bacteria in females rather than differences in initial bacterial exposure. Comparable studies using systemic bacterial challenge have likewise identified sex-specific differences in antibacterial immunity among hematophagous insects. Following bacterial injection into the hemocoel, female *Anopheles gambiae* cleared bacteria more efficiently than males [25], whereas male *Ae. aegypti* exhibited greater bacterial clearance than females under similar experimental conditions [23]. Similar patterns have also been reported in the non-hematophagous insect *D. melanogaster*, where males were more susceptible to systemic *Staphylococcus aureus* infection and harbored higher bacterial loads than females [26]. Together, these studies suggest that sex-specific differences in systemic antibacterial immunity may be common among insects, although the direction of these differences varies among species. The present study extends these observations to fleas by demonstrating that female *C. felis* has greater control of systemic bacterial infection compared with males.

The reduced bacterial burden observed in females was accompanied by a marked survival advantage following systemic infection, further supporting the conclusion that females possess more effective systemic immune defenses than males. While *E. coli* infection reduced survival in both sexes, the effect was substantially greater in males, with infected females nearly twice as likely to survive the 5-day observation period. Interestingly, sterile injury alone significantly reduced male survival but had little effect on females, suggesting that males are less capable of tolerating or recovering from disruption of the cuticle and entry into the hemocoel, even in the absence of bacterial challenge. This sex-specific effect of sterile injection on survival also highlights a limitation of the experimental model, as the procedure itself affected males and females differently. Additionally, because the flea cuticle was not surface sterilized prior to injection, the possibility that cuticle-associated microorganisms entered the hemocoel during injection and contributed to the response observed in the sterile-injury group cannot be excluded. Sex-biased survival following bacterial infection has been documented across insects, although the direction of this bias varies considerably depending on the host species, bacterial pathogen, host genotype, mating status, and environmental conditions [27,28,29,30,31,32,33,34,35]. Among hematophagous insects, Barletta Ferreira et al. [23] reported that female *Ae. aegypti* survived systemic bacterial infection better than males despite maintaining higher bacterial burdens, suggesting that female survival was associated with greater tolerance to infection rather than more efficient bacterial control. In contrast, female *C. felis* exhibited both lower bacterial burdens and greater survival, indicating that enhanced survival in fleas is associated with more effective control of systemic bacterial infection. Together, these findings demonstrate that the relationship between systemic infection and survival differs among hematophagous insects.

The greater ability of female *C. felis* to limit bacterial burdens within the hemocoel was accompanied by marked differences in circulating hemocyte abundance, suggesting that sex-specific variation in cellular immunity contributes to the enhanced antibacterial defenses observed in females. Regardless of treatment, females maintained nearly twice as many circulating hemocytes as males, indicating that sexual dimorphism in hemocyte abundance exists even in the absence of immune challenge. Similar female-biased differences have been reported in mosquitoes [25,36], scorpionflies [37], moths [38], and beetles [39]; however, studies in *D. melanogaster* have reported female-biased, male-biased, or no differences in adult hemocyte abundance [27,30,40], suggesting that baseline sexual dimorphism in circulating hemocyte populations varies among insect taxa. Moreover, female fleas responded to both sterile injury and bacterial infection by significantly increasing the number of circulating hemocytes, whereas males exhibited no detectable change. Similar increases in circulating hemocyte abundance following systemic bacterial challenge have been reported previously in female *C. felis* [10], but the present study demonstrates that this inducible response is largely restricted to females. Although few studies have examined sex-specific changes in circulating hemocyte abundance following infection, several studies demonstrate that hemocyte regulation differs between male and female insects. In the firebug *Pyrrhocoris apterus*, females increased circulating hemocyte numbers in response to changes in photoperiod, whereas males did not [41]. Likewise, hemocyte concentrations increased with age in the European earwig, although the increase was more pronounced in males than females [42]. Comparable differences in inducible hemocyte responses have also been observed among mosquitoes, where systemic bacterial infection increased circulating hemocyte abundance in *An. gambiae* but not in *Ae. aegypti*; however, these comparisons were made between species rather than between sexes [43]. Importantly, the perfusion method used in the present study quantified circulating hemocytes but did not measure sessile hemocytes associated with tissues or the body wall. Thus, the observed increase in circulating hemocyte abundance cannot be assumed to represent an increase in the total number of hemocytes within the flea. The mechanisms responsible for the increased number of circulating hemocytes remain unclear and may include shifts in the distribution between sessile and circulating hemocytes, as well as proliferation of circulating cells. Mitosis of circulating hemocytes was visually documented in female *C. felis* by Muñoz et al. [10], while mobilization of sessile hemocytes into the circulation has been described in mosquitoes following systemic bacterial infection [44,45]. Collectively, these findings demonstrate that female *C. felis* maintain more hemocytes in circulation and exhibit a greater increase in circulating hemocyte abundance following systemic challenge than males, although whether these differences reflect changes in total hemocyte number, hemocyte distribution, or both remains unknown.

In addition to maintaining more circulating hemocytes, females also exhibited a significantly greater proportion of cells participating in phagocytosis, further strengthening their cellular immune response. Importantly, phagocytic activity was assessed by evaluating the first 100 hemocytes encountered from each flea, thereby standardizing comparisons between sexes regardless of differences in total circulating hemocyte abundance. In contrast, individual hemocytes internalized similar numbers of bacteria in both sexes, indicating that the phagocytic capacity of individual cells does not differ between male and female fleas. Similar variability in phagocytic responses has been reported in other insects. In scorpionflies, females exhibited greater overall phagocytic activity than males because they possessed more circulating hemocytes and a higher proportion of cells engaged in phagocytosis, whereas the phagocytic capacity of individual hemocytes did not differ between the sexes [37]. This pattern closely parallels the results of the present study, in which females exhibited a greater proportion of phagocytic hemocytes despite no difference in the number of *E. coli* ingested per phagocytic cell. By comparison, male *D. melanogaster* hemocytes exhibit a slightly higher ex vivo phagocytic index than those of females [30], whereas adult female *An. gambiae* hemocytes carry a greater phagocytic burden than larval hemocytes, demonstrating that phagocytic activity can also vary across developmental stages [46]. Collectively, the lower bacterial burdens observed in female *C. felis* are associated with both a larger circulating hemocyte population and a greater proportion of hemocytes participating in phagocytosis, rather than greater bacterial uptake by individual phagocytic cells.

One factor that may contribute to the higher baseline hemocyte abundance observed in females is the tendency for female fleas to be larger than males [47]. Because females generally possess a greater body size and, presumably, a larger hemolymph volume, they may support a larger circulating hemocyte population; however, these factors were not evaluated in the present study. Even so, body size alone is unlikely to explain the observed sex-specific immune responses. Females not only maintained a larger circulating hemocyte population under naïve conditions but also uniquely increased circulating hemocyte abundance following injury and bacterial challenge while exhibiting a greater proportion of hemocytes participating in phagocytosis. Collectively, these findings indicate that although body size may contribute to baseline differences in circulating hemocyte abundance, it is unlikely to fully account for the sex-specific differences in cellular immune responses observed following challenge.

An additional factor that may have influenced immune responses is the reproductive status of the fleas. Adult *C. felis* begin feeding immediately after acquiring a host, mate shortly thereafter, and females become reproductively active within the first few days [48,49]. Consequently, the two- to three-day-old fleas used in this study were likely reproductively active during the experiments. Reproductive status has been shown to influence immune function in insects, although the magnitude and direction of these effects vary considerably among species and immune traits [27,34,35,42,50,51,52,53,54,55]. Whether reproductive status contributes to the sex-specific differences in systemic immunity observed in *C. felis* remains unknown and warrants further investigation.

In conclusion, this study demonstrates that male and female *C. felis* differ in their responses to experimental systemic bacterial challenge. Female fleas exhibited lower bacterial prevalence and burdens, greater survival, higher circulating hemocyte abundance, and a greater proportion of phagocytic hemocytes than males. These findings identify sex as a biological variable associated with variation in systemic antibacterial responses in cat fleas. Because bacteria were introduced directly into the hemocoel, the present study does not determine whether comparable differences occur following natural acquisition of flea-borne microorganisms. Future studies comparing male and female fleas following exposure through an infectious blood meal will be necessary to determine whether sex influences pathogen establishment, dissemination, or transmission.

## Data Availability

The data supporting the findings of this study are available within the article. Raw data are available from the corresponding author upon request.

## Abbreviations

CFU	Colony Forming Units
GFP	Green Fluorescent Protein
PBS	Phosphate-Buffered Saline
SEM	Standard Error of the Mean
TSA	Tryptic Soy Agar
TSB	Tryptic Soy Broth
